# The Digital Health: From the Experience of the COVID-19 Pandemic Onwards

**DOI:** 10.3390/life12010078

**Published:** 2022-01-06

**Authors:** Daniele Giansanti

**Affiliations:** Centro Tisp ISS, 00161 Rome, Italy; gianslele@gmail.com or daniele.giansanti@iss.it

Digital health has a long history of development and is particularly resonant in the last two years, due to the pandemic [1,2]. A recent study [2] revised the definitions associated with digital health. The authors undertook a quantitative analysis and term mapping of the published definitions of digital health. They analyzed 95 unique definitions of digital health, from both scholarly and general sources. The findings showed that digital health, as has been used in the literature, is more concerned about the provision of healthcare rather than the use of technology. The wellbeing of people, both at population and individual levels, have been emphasized more than the care of patients suffering from diseases. Furthermore, the use of data and information for the care of patients was highlighted. A dominant concept in digital health appeared to be mobile health (mHealth), which is related to other concepts, such as telehealth, eHealth, and artificial intelligence in healthcare.

Even the World Health Organization (WHO) entered into this discussion [3]. The WHO is harnessing the power of digital technologies and health innovation to accelerate the global attainment of health and well-being. WHO has three key objectives [4] to promote the adoption and scale-up of digital health and innovation:

- Translating the latest data, research, and evidence into action: this means promoting standards for interoperability and data sharing, and supporting the implementation of digital solutions that contribute to informed decision making;
- Enhancing knowledge through scientific communities of practice;
- Systematically assessing and linking the needs of the country with the supply of innovations.

National and supranational entities are also clearly addressing this issue. For example, the Food and Drug Administration (FDA) [5] in the U.S.A., states that digital health includes categories, such as mHealth, health information technology (IT), wearable devices, telehealth and telemedicine, and personalized medicine. Furthermore, the FDA recognizes that (a) from mobile medical apps and software that support the clinical decisions that doctors make every day to artificial intelligence and machine learning, digital technology has been driving a revolution in health care. (b) Digital health tools have the vast potential to improve our ability to accurately diagnose and treat disease and to enhance the delivery of healthcare for the individual. (c) Digital health technologies use computing platforms, connectivity, software, and sensors for healthcare and related uses; these technologies span a wide range of uses, from applications in general wellness to applications as a medical device.

The potential benefits have been identified:

- Reduce inefficiencies,
- Improve access,
- Reduce costs,
- Increase quality, and
- Make medicine more personalized for patients.

The following FDA action topics were also highlighted in the context of digital health, to provide clarity using practical approaches that balance the benefits and risks:

- Software as a Medical Device (SaMD);
- Artificial Intelligence and Machine Learning in Software as a Medical Device;
- Cybersecurity;
- Device Software Functions, including Mobile Medical Applications;
- Health IT;
- Medical Device Data Systems;
- Medical Device Interoperability;
- Telemedicine;
- Wireless Medical Devices.

As a supranational entity, the European (Eu) Commission has also addressed the issue [6,7]. It stated that digital health is based on tools and services that use information and communication technologies (ICTs) to improve prevention, diagnosis, treatment, monitoring, and management of health-related issues, and to monitor and manage lifestyle habits that impact health. Digital health and care is innovative and can improve access to care and the quality of that care, as well as increase the overall efficiency of the health sector. It identified the following Eu pillars:

- Pillar 1: Secure data access and sharing;
- Pillar 2: Connecting and sharing health data for research, faster diagnosis, and improved health;
- Pillar 3: Strengthening citizen empowerment and individual care through digital services.

WHO’s objectives in launching the integration of digital health, the FDA actions topics, and the EU pillars are shareable and are all elements put to the test in the last two years, due to the COVD-19 pandemic.

From a quick overview on Pubmed using the search key “digital health” [8], we observe that, at the date of writing this editorial (28 December 2022), there are 41,165 studies that have addressed this issue.

We also observe that, in the last two years, marked by the pandemic, we have seen the publication of 18446 works, equal to 44.32% of the total.

Several studies and insights published during 2020–2021 have developed these issues, analyzing the advantages and disadvantages, successes and failures, and offering reflections on the implications and issues of these technologies in the health domain. The results of these investigations will affect the redesign of hospital and outpatient management, based on digital innovation using eHealth and mHealth. It has been highlighted by the WHO, FDA, and EU [4,5,6,7], that digital health encompasses a broad spectrum of technologies, including wearable personal devices and internal devices, as well as various types of sensors and innovative solutions. Digital health can help in the diagnosis, treatment, and monitoring of health conditions, offering new potential to both the population and the insiders of the health domain. During the pandemic, this approach made it possible to offer assistance and continue care at home, protecting patients, preserving health workers, limiting the spread of the virus, and reducing the need for hospitalization [9]. For example, the opportunity to make digital measurements of oxygen saturation [10] at home has been used to make fundamental decisions for the health of patients, such as the choice between hospitalization and respiratory support. It has also become possible to monitor frail patients from home (e.g., with diabetes or cardiovascular or oncological problems) [11], thus improving the continuity of care and reducing the pressure on the hospitals. Digital health also continues to contribute to the fight against the pandemic in various new ways, such as the management of digital contact tracing [12] and vaccination processes through smart technology [13]. Limitations were also clearly shown, which mainly concerned the following points:

- The digital divide;
- Organizational aspects (both with regard to administrative and technological aspects).

The digital divide has two important components. The first component is represented by the difficulty in accessing to the infrastructures; to date, this also remains a problem in the richest and most technologically advanced countries in the world [14]. The second component is represented by the literacy [15]. These two components of the digital divide were particularly visible during the COVID-19 pandemic [16,17,18,19,20,21]. They can depend on cultural, ethnic, social, national, and political factors [19,20]; furthermore, they can exacerbate the disparities [21].

In regards to the organizational aspects, it can be highlighted that they are broad-spectrum. Undoubtedly, developing countries start from a base of technological need that must exist before they can fully apply digital health in the health domain. However, even the most developed countries, such as Italy, found themselves facing difficulties, especially at an early stage, which, according to some authors [22], were caused by the following factors:

- The scattered distribution and heterogeneity of available tools;
- The lack of integration with the electronic health record of the national health system;
- The poor interconnection between telemedicine services operating at different levels;
- The lack of a real multidisciplinary approach to the patient’s management;
- The heavy privacy regulations and lack of clear guidelines, together with the lack of reimbursement.

From the Editorial, it emerges that the pandemic was an important driver for digital health. This concerned: (a) the improvement of medical and technological knowledge; (b) the stimulus for the implementation of solutions in the health domain; (c) the stimulus for the resolution of long-standing problems related to management and organizational aspects (e.g., reimbursement, and introduction into the treatment process); And (d) the proposition of new solutions (e.g., contact tracing). The analysis of how well it worked and how much it needs to be improved, is important to both improve the offer from the point of view of technology and quality, and to focus the interest of the scholars at 360°. All that is important to both continue the battle against COVID-19 and to prepare new effective and functioning stable health models for the post-pandemic future. For this purpose, the Special Issue, “The Digital Health in the Pandemic Era” (https://www.mdpi.com/journal/life/special_issues/DigitalHealth_Pademic (accessed on 28 December 2021)) [23], has been prepared with the aim of creating a meeting of experiences of the experts of the health domain.
